# Electrophysiological properties and seizure networks in hypothalamic hamartoma

**DOI:** 10.1002/acn3.51033

**Published:** 2020-04-16

**Authors:** Di Wang, Yongzhi Shan, Fabrice Bartolomei, Philippe Kahane, Yang An, Muyang Li, Huaqiang Zhang, Xiaotong Fan, Siqi Ou, Yanfeng Yang, Penghu Wei, Chao Lu, Yihe Wang, Jialin Du, Liankun Ren, Yuping Wang, Guoguang Zhao

**Affiliations:** ^1^ Department of Neurology Xuanwu Hospital Capital Medical University Beijing China; ^2^ The Beijing Key Laboratory of Neuromodulation Beijing China; ^3^ Department of Radiology Xuanwu Hospital Capital Medical University Beijing China; ^4^ Department of Neurosurgery Xuanwu Hospital Capital Medical University Beijing China; ^5^ Institut de Neurosciences des Systèmes Aix‐Marseille University Marseille France; ^6^ Service de Neurophysiologie Clinique Hôpital de la Timone AP—HM Marseille France; ^7^ Inserm U836 Grenoble France; ^8^ University Grenoble Alpes GIN Grenoble France; ^9^ Neurology Department CHU de Grenoble, Hospital Michallon Grenoble France; ^10^ Beijing Institute for Brain Disorder Beijing China; ^11^ Department of Pediatrics Xuanwu Hospital Capital Medical University Beijing China

## Abstract

**Objective:**

Little is known about the intrinsic electrophysiological properties of hypothalamic hamartoma (HH) in vivo and seizure network since only few cases using stereoelectroencephalography (SEEG) electrodes exploring both cortex and HH have been published. To elucidate these issues, we analyzed simultaneous SEEG recordings in HH and cortex systematically.

**Methods:**

We retrospectively investigated data from 15 consecutive patients with SEEG electrodes into the HH for the treatment purpose of radiofrequency thermocoagulation treatment. Additional SEEG electrodes were placed into the cortex in 11 patients to assess extra‐HH involvement. Interictal discharges within the HH and anatomo‐electroclinical correlations during seizures of each patient were qualitatively and quantitatively analyzed.

**Results:**

Overall, 77 electrodes with 719 contacts were implanted, and 33 spontaneous seizures were recorded during long‐term SEEG monitoring. Interictally, distinct electrophysiological patterns, including isolated intermittent spikes/sharp waves, burst spike and wave trains, paroxysmal fast discharges, periodic discharges, and high‐frequency oscillations, were identified within the HH. Notably, synchronized or independent interictal discharges in the cortex were observed. Regarding the ictal discharges, the electrical onset pattern within the HH always started with abrupt giant shifts superimposed on low‐voltage fast activity across patients. The gelastic seizure network mainly involved the HH, orbitofrontal areas, and cingulate gyrus. Seizures with automatisms and impaired awareness primarily propagated to mesial temporal lobes. Moreover, independent ictal discharges arising from the mesial temporal lobe were detected in three out of nine patients.

**Interpretation:**

This study comprehensively reveals intrinsic electrophysiological patterns and epileptogenic networks in vivo, providing new insights into the mechanisms underlying cortical and subcortical epileptogenesis.

## Introduction

Hypothalamic hamartomas (HHs) are rare developmental malformations typically arising from the tuber cinereum. They have been drawing considerable interest because of a range of neurological symptoms, neuropsychiatric comorbidities and endocrine disturbances.^1^, ^2^ In particular, epilepsy has been recognized as the hallmark clinical picture of HH, characteristically presenting with drug resistant gelastic/dacrystic seizures often associated with other types of seizures.^3^, ^4^ It is well known that noninvasive electroencephalography (EEG) monitoring is unproductive and misleading in patients with epilepsy and HH. Electrophysiological properties characterizing HH epileptogenicity in vivo have rarely been investigated.^5^ A first study using intracranial EEG recordings^6^ with electrodes targeting only the cortex in eight patients with HHs revealed focal seizure‐onset activity in the cortical area, but cortical resection failed to control seizures. However, subsequent stereoelectroencephalography (SEEG) studies, including four cases from Grenoble,^7^, ^8^, ^9^ two cases from Marseille,^10^ and one case from London,^11^ have explored various cortical regions and HH, demonstrating the inherent epileptogenicity of HH in gelastic seizures (GS), as well as more complex patterns of epileptogenicity, particularly affecting the cortical regions in other kind of seizures.^10^ Two other case reports,^4^, ^12^ based on two‐step invasive EEG studies, have revealed the primary role of HHs in GS genesis. Electrophysiological data, together with functional neuroimaging findings and prolonged seizure remission after surgical management of HH in patients with epilepsy, indicate that HHs represent an important and unique human epileptic entity of subcortical origin.^12^, ^13^, ^14^, ^15^

Despite the demonstration of the critical role of HHs in seizure onset, the knowledge of the intrinsic electrophysiological properties and epileptogenic network related to HHs in vivo is quite incomplete since only few cases using SEEG electrodes exploring both cortex and HH have been published as indicated above. To date, numerous studies have investigated the electrophysiological characteristics of cortical epileptogenic tissue. A variety of cortical interictal patterns including trains of repetitive, rhythmic and periodic spikes/sharp waves, bursting epileptiform activity, “brushes,” and high‐frequency oscillations (HFOs) have been described over the few decades besides classical interictal discharges manifesting as a spike, spike, and wave or sharp wave.^16^, ^17^ Also, distinct ictal patterns of epileptic tissue were identified during seizures arising from cortical lesions.^18^, ^19^ Moreover, considering that focal seizures consist of coordinated activity across distributed areas, thus research on epilepsy as a dynamic process over a pathological network of interconnected brain regions has increased appreciably.^20^, ^21^, ^22^ Undoubtedly, these findings from cortical tissue deep the understanding of the pathophysiology of epilepsy and have implication of developing novelty clinical diagnostic methods. Nevertheless, the above mentioned multiple anomalous discharges as well as network mechanisms of the distinct semiology related to subcortical hamartoma is far less documented mainly limited by poorly informative scalp EEG, no animal model for epilepsy with HH and the scarcity of direct recording data in vivo.^10^, ^23^, ^24^

Seizures related to HHs are usually pharmacoresistant and resective surgery of the HH can be effective but is associated with a non‐negligible rate of complications.^14^ Although several lesioning techniques for HHs have been proposed, the use of SEEG‐guided radiofrequency‐thermocoagulation (RF‐TC) is increasingly popular due to its high effectiveness and low complication rate. In our hospital, we have developed a multidisciplinary team (MDT) for the treatment of HH‐associated pharmacoresistant epilepsy that privileges the use of SEEG‐guided RF‐TC.^25^ All the patients underwent SEEG electrode implantation into the primary target of the HH. Extra‐HH SEEG electrodes were designed if a cortical seizure onset zone was suspected. Herein, we describe SEEG recordings of a series of 15 patients with HHs. The aim of the study was twofold: to assess the intrinsic interictal charge pattern within the HH and the correlation with cortical activity; to study the ictal discharge pattern and firstly quantify the HH‐cortical network underlying distinct seizure semiology.

## Materials and Methods

### Patients

A series of 15 patients (6 females, 18 ± 9.4 years of age, age range: 6–37 years old; mean duration of epilepsy: 12.5 ± 9.3 years, duration range: 1–36 years) with an HH presenting with refractory focal seizures were retrospectively studied between July 2015 and June 2018 at Xuanwu Hospital, Capital Medical University, Beijing, China. The diagnosis of HH was based on T1‐weighted, T2‐weighted, and fluid‐attenuated inversion recovery magnetic resonance imaging (MRI). Table S1 summarizes the clinical profiles of all 15 patients in which extraoperative recordings were performed. The treatment outcome of RF‐TC on HH for eight of these patients has been previously reported.^25^

All patients underwent the same protocol of presurgical evaluation, including detailed history, seizure semiology, routine MRI, video scalp EEG, or positron emission tomography. The HH were categorized by their location within the hypothalamus as right/left by the asymmetry of attachment or location. The implantation of SEEG electrodes was scheduled for the treatment of SEEG‐guided RF‐TC. The primary target was the HH in all patients. Moreover, considering that extra‐HH seizure‐onset zones have been frequently reported in prior studies, the anatomo‐electroclinical hypothesis was further individually formulated by our MDT. Therefore, plans for extra‐HH SEEG electrodes, including the number of electrodes and the sites of implantation, were designed if the cortical seizure‐onset zone and early propagation area specific to each patient were presumed.

This study was approved by the Institutional Review Board Committee at Xuanwu Hospital and conducted in accordance with the ethical standards of the Declaration of Helsinki.

### Implantation of SEEG electrodes and SEEG recording

Multilead SEEG electrodes (Alcis, Besancon, France) with 5, 8, 10, or 12 contacts were 2 mm in length, 0.8 mm in diameter, and 1.5 mm apart. Magnetic resonance venography and magnetic resonance angiography sequences (3.0 T, Siemens) were also obtained and fused to avoid major vessel injury in the design of electrode trajectories. All 15 patients underwent frameless depth electrode implantation with the guidance of Robotic Stereotactic Assistance (Medtech, Montpellier, France) using oblique trajectories. Finally, the patient underwent a CT or MRI scan after electrode implantation to verify the exact location of each electrode and to check for postoperative complications.

After implantation, all patients underwent long‐term video monitoring to capture habitual clinical seizures. The signals of patients 2 and 3 were recorded by the Micromed EEG data acquisition system with a broadband sampling frequency from 0.01 to 256 Hz. The other patients were recorded using the Nicolet system with a broadband sampling frequency from 0.01 to 2048 Hz. All the recordings were acquired and referenced to a common contact placed subcutaneously.

### Reconstruction of SEEG electrodes in the brain

Depth electrodes of each patient were reconstructed within Montreal Neurological Institute (MNI) space using Lead‐DBS software (://www.lead-dbs.org) following the previously described protocol.^26^ Briefly, postoperative CT was linearly coregistered to preoperative MRI using SPM12 (http://www.fil.ion.ucl.ac.uk/spm/software/spm12/; postoperative MRI) or BRAINSFit software (https://www.nitrc.org/projects/multimodereg/; postoperative CT). Coregistrations were manually controlled for each patient and refined if needed. Then, the images were normalized into ICBM 2009b NLIN asymmetric space using the SyN approach implemented in advanced normalization tools (http://stnava.github.io/ANTs/) based on the preoperative MRI. The HH of all patients in the MNI coordinate space was divided into regions along its anteroposterior axis with previously described methods.^27^

All electrodes were color‐coded and represented in the transparent three‐dimensional brain surface.

### Data analysis

SEEG activities in this study were evaluated using the bipolar montages between contiguous contacts to highlight the focal voltage changes. Notably, the voltage of local field potentials within the HH was rescaled to be comparable to cortical activity by visual inspection. The visual inspection of the SEEG data was performed blinded by experienced neurophysiologists (D.W. and L.R.) for qualitatively detecting and categorizing multiple anomalous discharges. Patterns were only considered to be present when consensus was achieved between the two independent reviewers. The quantitative method was used in combination with the qualitative analysis. The electrophysiological data were processed using EEGLAB and customized MATLAB codes (MathWorks Inc., Natick, MA) unless otherwise stated.

### Analysis of interictal discharges

Regarding the interictal discharges, 4 h of SEEG without seizures for 2 h prior to or after the segments chosen were selected for analysis. The interictal biomarkers from epilepsy patients with epileptogenic cortical lesions as defined in prior publications, including stereotyped, isolated intermittent sharp/spike waves, polyspikes, trains of rhythmic spikes/sharp waves, paroxysmal fast activities, periodic or quasiperiodic discharges, and delta brush of slow waves imposed with fast activities were screened in all 15 patients. HFOs were identified on zoomed‐in SEEG data, and the spectrograms were performed to display transient HFOs using continuous wavelet transform.^28^, ^29^, ^30^

The hamartoma was investigated in all cases, and various cortical areas were also evaluated in 11 of the 15 patients. The correlations of interictal discharges between the HH and cortex were further assessed in 11 patients. Synchronized patterns were considered with the simultaneous appearance of interictal discharges in both the HH and cortex. Otherwise, interictal discharges that occurred within only the HH or cortex only were regarded as independent activities.

### Analysis of anatomo‐electroclinical correlations

The video‐SEEG data of all recorded clinical seizures of each patient were reviewed. Epileptic seizure types were generally defined according to the 2017 ILAE classification.^31^, ^32^ Based on the consensus description, GS referred to epileptic events presenting with bursts of laughter, usually without an appropriate affective tone. Since these are generally considered to be variations of the same seizure manifestation, GS was not further classified in the study. Ictal discharges were evaluated in the context of the broadband frequency band (0.01–250 Hz) to highlight the slow activity. Unless stated otherwise, SEEG data were displayed in the conventional frequency band at 0.5–70 Hz.

To address the epileptogenic network of distinct seizure semiology, the analysis of anatomo‐electroclinical correlations was conducted. For distinct seizure semiology, the time window from the electrical onset to the emergence of all the semiological elements was selected by visual inspection. The subset of anatomic structures that were significantly engaged during the time window was considered to be involved in such a distinct seizure network. The structures without EEG modifications were defined as noninvolved.^33^ All the involved and noninvolved areas were displayed on the standard MNI template.

In addition, cortico‐cortical evoked potentials (CCEP) were conducted to track the HH‐cortical connections in vivo.^34^, ^35^ In each session of the present study, 60 stimuli were delivered to two adjacent contacts within HH using the Nicolet system. Each stimulus consisted of a pulse frequency of 1 Hz, a constant current square wave pulse of 0.3 msec duration with alternating polarity and current intensity of 2 mA. CCEP were averaged time‐locked to the onset of each electrical stimulus off‐line with a time window of −200 to +400 msec and a low‐frequency filter of 1.0 Hz and a high‐frequency filter of 40 Hz, which was described previously, were averaged.^36^ The appearance of N1 component within 50 msec after stimulation reflected connectivity between the two sites.

### SEEG‐guided radiofrequency thermocoagulation (RF‐TC)

SEEG‐guided RF‐TC was performed based on the findings of chronic SEEG monitoring in these patients. Technically, RF‐TC lesions were created by the methods previously described.^25^ Outcomes were acquired through telephone calls and outpatient visits and further categorized according to Engel's classification.

### Statistical analysis

In the analysis of the potential links between seizure‐onset origin and the onset age of epilepsy, since the sample size is very small, no statistical inference tests were performed. To assess the influence of seizure‐onset origin on postoperative prognosis, patient numbers with different surgical outcomes from different onset origins are presented with bar graphs. To show the influence of hypothalamic regions on seizure types, patient numbers with differing seizure types in different hypothalamic regions are presented with bar graphs. For the numerical data including seizure onset age and duration, error bar charts with means and standard deviations of the data were graphically displayed. All analyses were performed using customized MATLAB codes.

### Data Availability Statement

The data that support this study are available on request. The data are not publicly available as they contain information that could compromise research participant privacy consent.

## Results

### Patient characteristics

Ten patients presented with GS followed by additional seizure types during the clinical course, three patients with only GS and two patients with only impaired awareness seizures. Two patients had undergone previous Gamma Knife surgery, one patient had previously undergone open surgery, and one patient had undergone both Gamma Knife surgery and open surgery.

**Figure 1:**
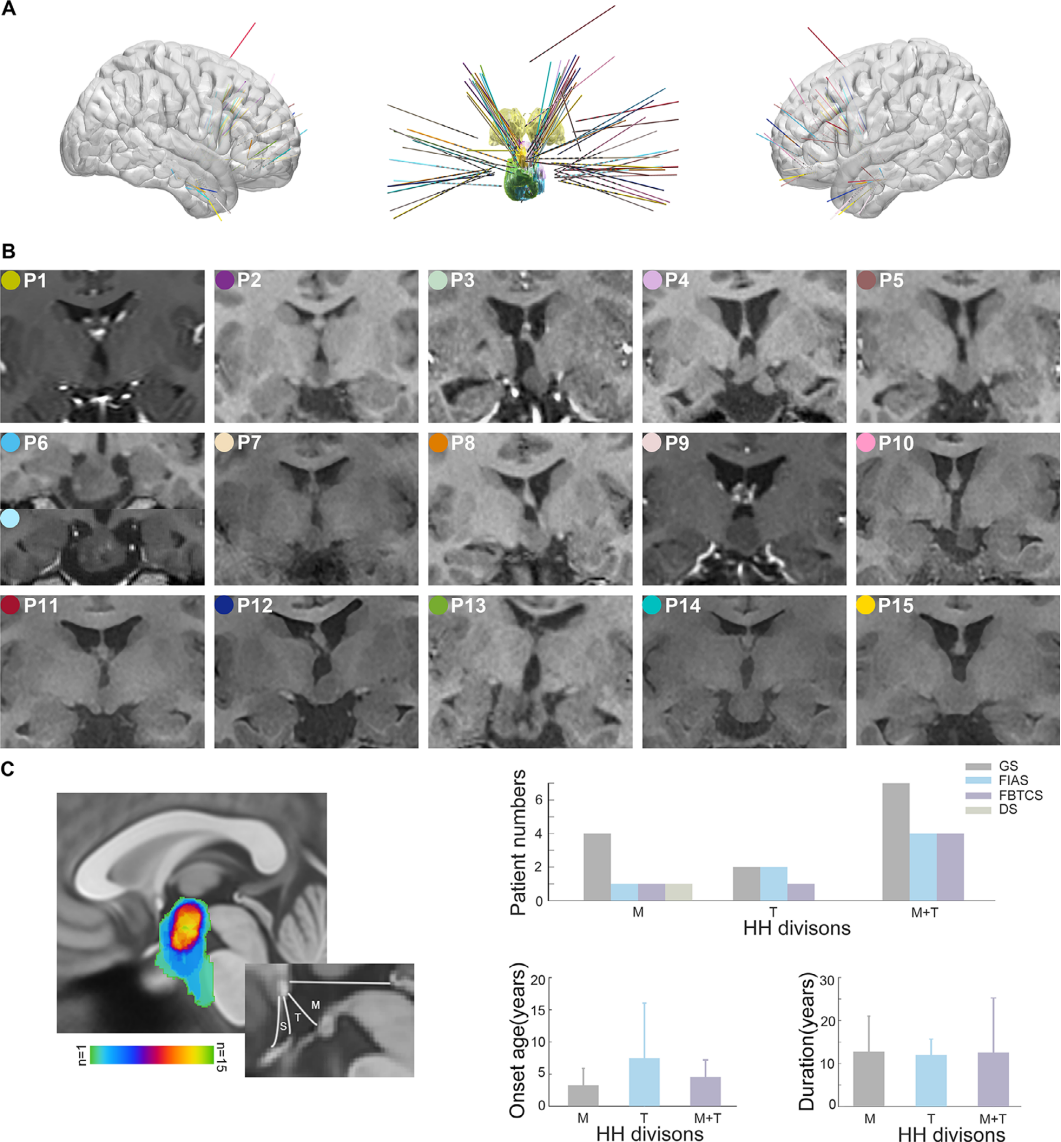
Reconstructions of SEEG electrodes in all fifteen patients and individual anatomical locations of the HHs. (A) Right (left) and left (right) views of three‐dimensional brain surface superimposed with all patients' SEEG electrodes in MNI coordinate space. A different color identifies each patient's electrodes consistent with the color shown in (B). (Middle panel) The trajectories of depth electrodes into the HH overlaid with all patients. The yellow color labels the bilateral thalamus in the coronal view and the HHs are color‐coded corresponding to patient‐specific electrodes. (B) Coronal views of individual anatomical HHs in 15 patients. Note that patient 6 has undergone RF‐TC surgery twice. (C) HH location overlaid in MNI space and the correlation with clinical profiles. The hypothalamus is commonly divided into the supraoptic (S) region, mammillary (M) region, and tuberal (T) region along its anteroposterior axis. SEEG, stereoelectroencephalography; HHs, hypothalamic hamartomas; MNI, Montreal Neurological Institute; RF‐TC, radiofrequency‐thermocoagulation.

In total, 77 electrodes with 719 contacts were implanted in 15 patients (including 2 electrodes with 16 contacts during the second SEEG implantation of patient 6), in which 39 electrodes with 299 contacts were implanted in HH. In six patients, the bilateral cortical areas were explored, and in five patients, only cortical areas ipsilateral to the attachment of the HH were explored. All the individual reconstructions of electrodes and anatomical HH locations are shown in Figure 1.

As shown in Figure 1C, the HH connected to the hypothalamus in the posterior region, including the mammillary region (M) in four patients, tuberal region (T) in four patients and mammillary region plus tuberal region (M + T) in seven patients. The patients with HH connecting to the posterior hypothalamus in the M region were tended to have the earliest onset age of epilepsy.

### Interictal SEEG findings

**Figure 2:**
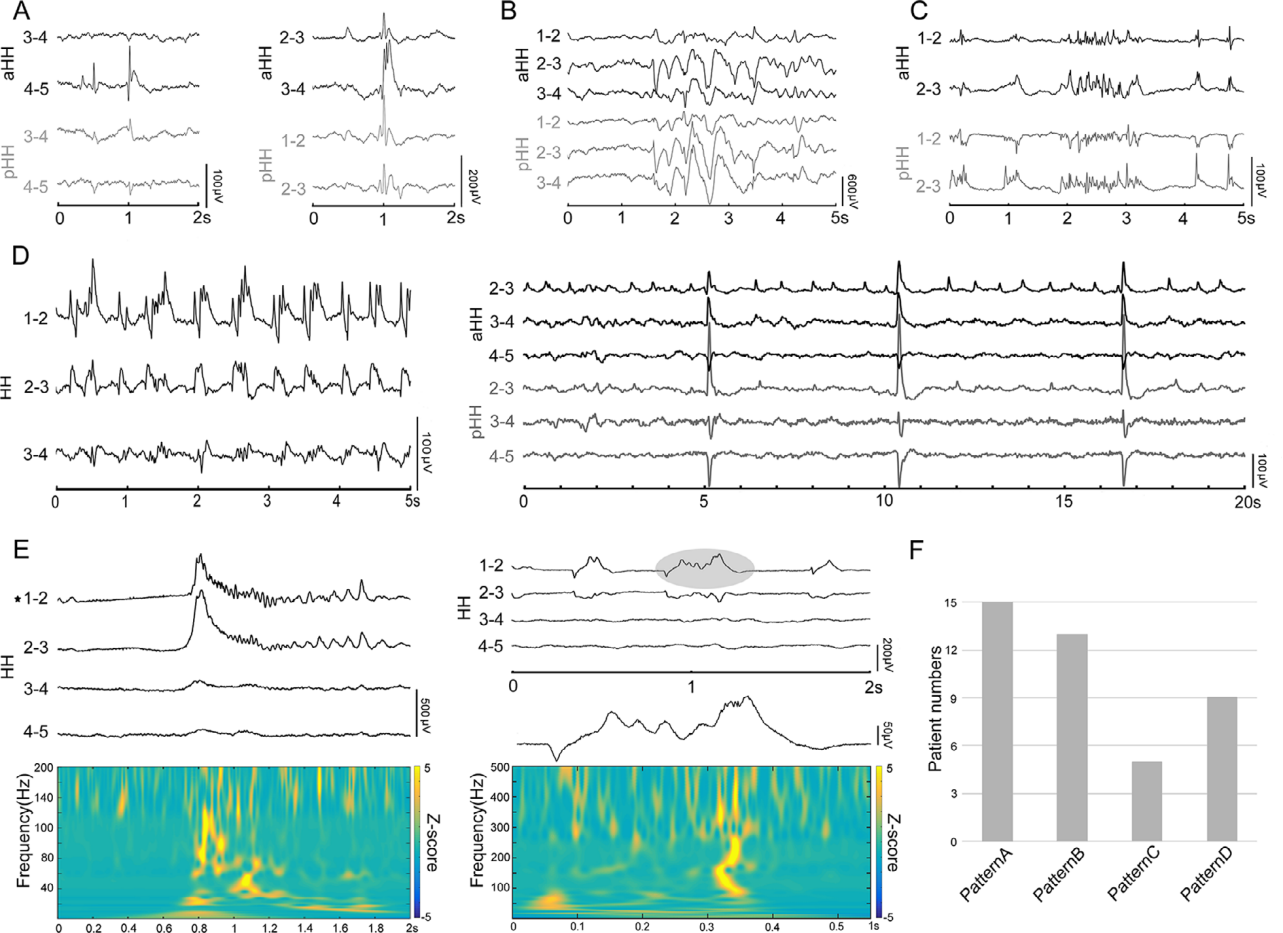
Intrinsic interictal discharge patterns inside HHs. Black and gray color‐coded LFPs indicate the aHH and pHH recordings, respectively. (A) Isolated intermittent spikes/sharp and waves (left; patient 8) and polyspikes (right; patient 4). (B) Burst spike and wave trains (patient 3). (C) Paroxysmal fast discharges (patient 14). (D) Periodic discharges. Delta brush consisting of 8–25 Hz spindle‐like, rhythmic activity superimposed on 0.5–2 Hz delta waves (left, patient 10). Periodic discharges of 1–2 Hz and slow periodic discharges of 0.2 Hz (right, patient 2). (E) Ripples (80–200 Hz; left) and nested ripples and fast ripples (200–500 Hz; right) displayed with raw SEEG and spectrograms. (F) The detection of interictal discharge patterns in all patients. Patterns A, B, C, and D refer to interictal discharges in (A–D), respectively. HHs, hypothalamic hamartomas; LFPs, local field potentials; aHH, anterior HH; pHH, posterior HH; SEEG, stereoelectroencephalography.

Heterogeneous interictal discharges were identified within the HH, which were further categorized into four distinct discharge patterns based on morphology and rhythmicity. Spikes or sharp waves (pattern A) occurring sporadically and intermittently were detected in all 15 patients (Fig. 2A). Burst spike and wave trains (pattern B) were highly prevalent in 13 patients (Fig. 2B). Fast discharge trains lasting >3 sec at beta frequency bands ranging from 13 to 20 Hz (pattern C) were frequently detected in 5 patients (Fig. 2C). Interestingly, interictal discharges showed a tendency to periodically recur (pattern D) in 9 patients. Periodic spikes/sharp waves were observed in eight patients, and one patient displayed a periodic delta brush with the appearance of rhythmic delta waves at 0.5–2 Hz with superimposed 0.5 sec‐bursts of 8–25 Hz activity (Fig. 2D). In particular, as a promising biomarker of epileptogenicity, spontaneous HFOs, including ripples (80–200 Hz) and fast ripples (200–500 Hz), were also identified from the interictal investigation inside the HHs (Fig. 2E). For each patient, multiple patterns were detected. The analysis showed that five patients had two patterns, eight patients had three patterns, and two patients had four patterns.

**Figure 3:**
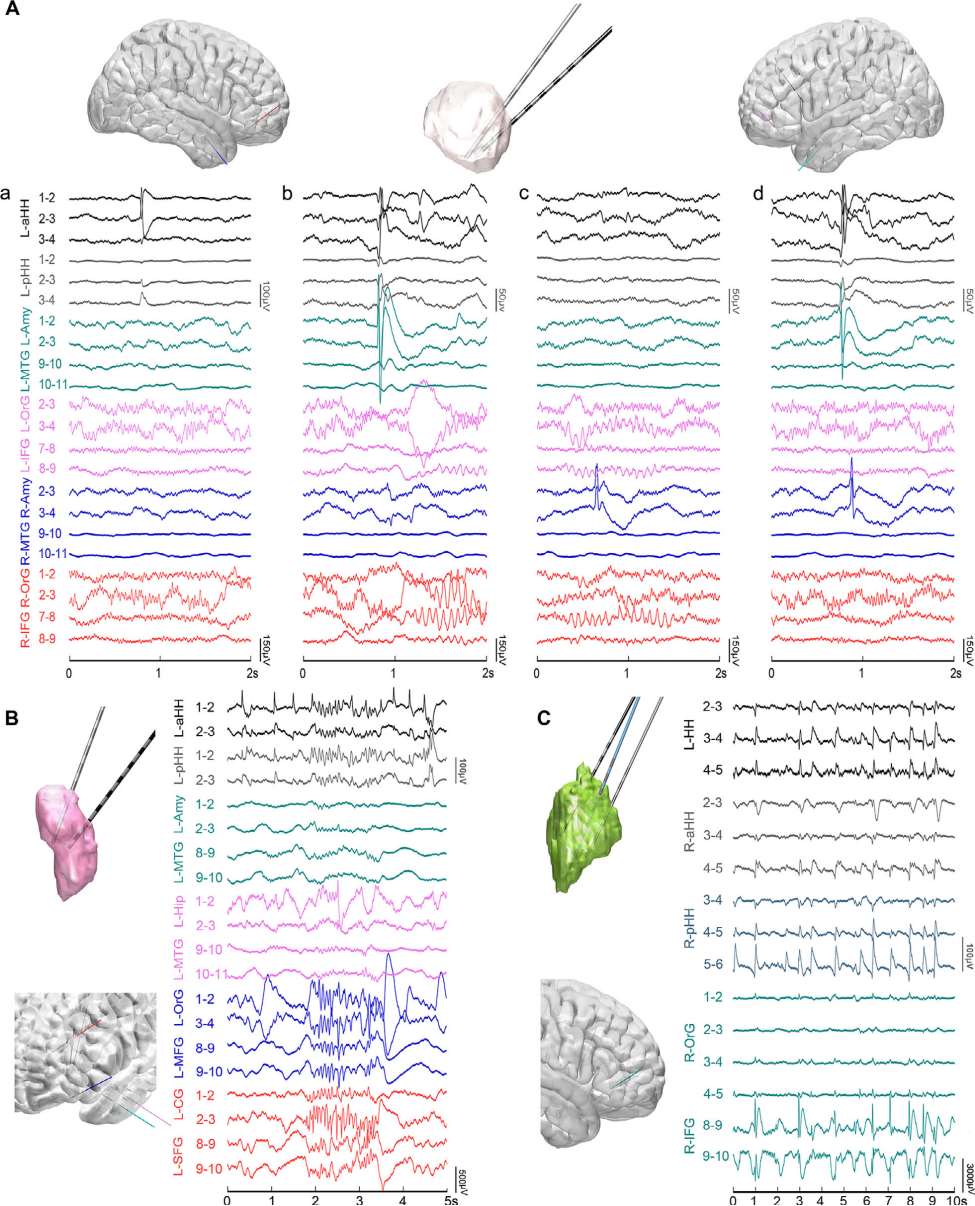
The correlation of interictal discharges between the HH and extra‐HH areas. Notably, the voltage of LFPs inside the HH and cortex are differentially scaled. (A) (Patient 9) The color‐coded reconstruction of depth electrodes into the right brain (top left), HH (top middle) and left brain (top right) consistent with the colors of the SEEG traces. (a) Isolated interictal discharges only inside HH. (b) Almost simultaneous discharges both inside the HH and ipsilateral amygdala. (c) Isolated interictal discharges only in the amygdala. (d) Interictal discharges in the contralateral amygdala after simultaneous discharges inside the HH and ipsilateral amygdala. (B) Synchronous paroxysmal fast discharges across the HH and neocortex (patient 15). (C) Synchronous rhythmic spikes/sharp waves across the HH and prefrontal cortex (patient 13). HH, hypothalamic hamartoma; LFPs, local field potentials; SEEG, stereoelectroencephalography.

Furthermore, interictal discharges were detected within extra‐HH areas in all 11 patients with cortical electrodes. However, the correlation between interictal discharges inside and outside the HH varied, including isolated interictal discharges only within the HH, independent interictal discharges within the extra‐HH areas and synchronous interictal discharges in the HH and extra‐HH areas (Fig. 3A). Additionally, fast discharges at the beta frequency band (pattern C) and periodic discharges (pattern D) within the HH, as shown in Figure 3B and C, respectively, were concurrently observed in the hippocampus, amygdala, and neocortex. The lateralization of the interictal discharges was always ipsilateral to the predominant side of the hamartoma.

### Ictal pattern and seizure network

Thirty‐three focal spontaneous seizures were recorded in 13 patients, which were classified into three types of seizure types: GS, focal impaired awareness seizure (FIAS), and focal to bilateral tonic–clonic seizure (FBTCS). Among these seizures, 20 were GS including seizures presenting with laughing or the appearance of laughing as an early prominent feature followed by tonic seizures, 11 were FIAS and 2 were FBTCS. The GS was recorded in eight patients, including one patient having both GS and automatisms with FIAS recorded, FIAS was recorded in four patients that was followed by GS in one patient, and FBTCS was recorded in two patients.

**Figure 4:**
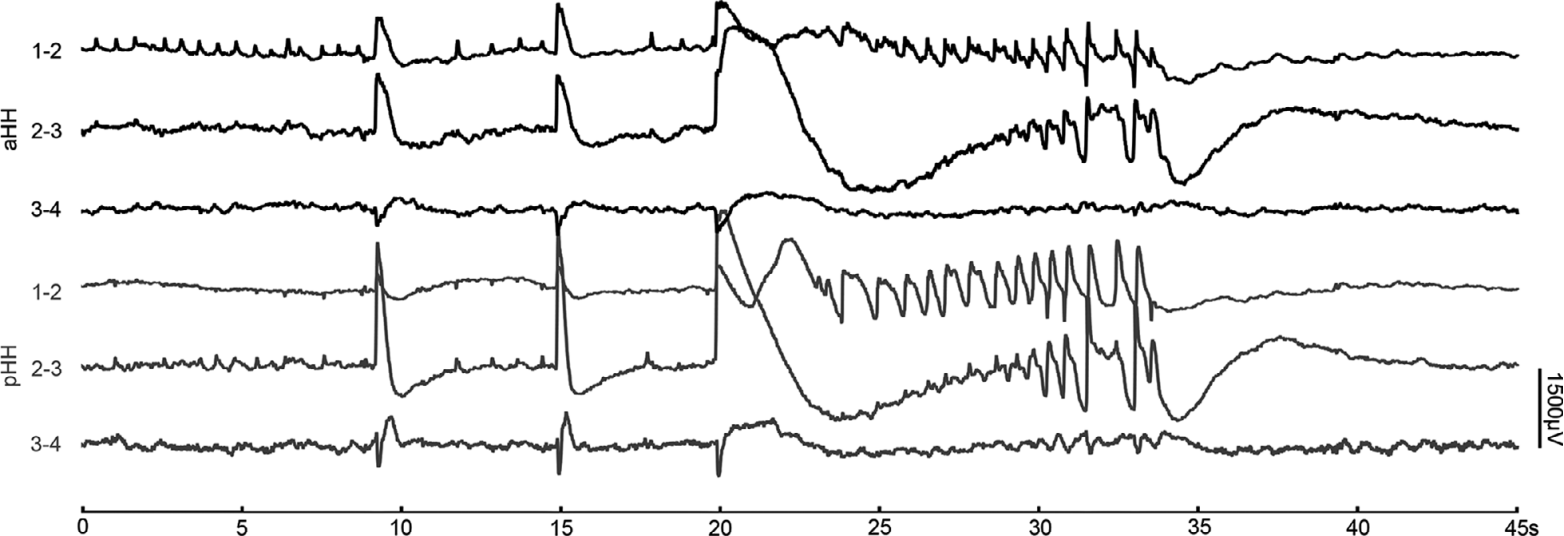
Intrinsic seizure onset pattern inside HH (Patient 2). Peri‐ictal SEEG epoch of broadband frequency recordings from 0.01 to 200 Hz showing pre‐ictal discharges, followed by abrupt giant slow shifts superimposed with low‐voltage fast activity. HH, hypothalamic hamartoma; SEEG, stereoelectroencephalography.

Despite different seizure semiology, the ictal discharges at seizure onset within the HH were similar. The ictal pattern was always characterized by abrupt giant direct current shifts superimposed with low‐voltage fast activity in the beta‐gamma range that could be preceded by variable numbers of preictal discharges with high amplitude (Fig. 4).

**Figure 5:**
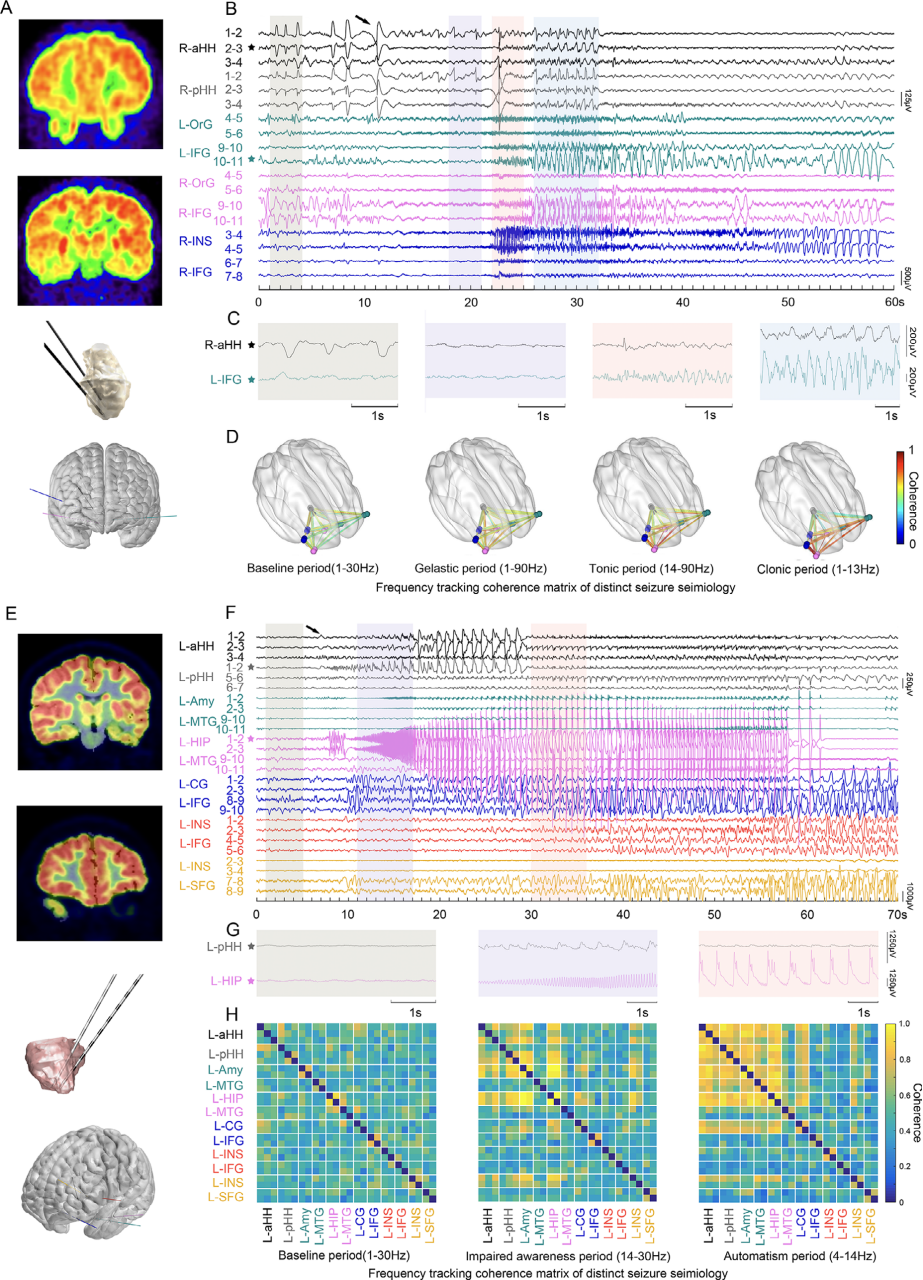
The HH‐cortical propagation of ictal discharges underlying prominent gelastic seizure (Patient 7) and automatisms with impaired awareness (Patient 5). (A) FDG‐PET show focal hypometabolism in bilateral orbitofrontal cortex and right insula which are targeted by extra‐HH electrodes. (B) The black arrow indicates the electric onset inside HH. Representative baseline period (1–4 sec), gelastic period (18–21 sec), tonic period (22–25 sec), and clonic period (26–32 sec) are shaded in semi‐transparent grey, purple, pink and blue respectively. (C) Magnified representative LFPs inside HH (R‐aHH 2–3, black star) and left inferior frontal gyrus (L‐IFG 10–11, green star) during the four periods that are demonstrated in (B). (D) Frequency‐tracking cross‐coherence networks of the four periods. (E) FDG‐PET show focal hypometabolism on left medial temporal lobe, insula, and subgenual area which are targeted by extra‐HH electrodes. (F) The black arrow indicates low amplitude fast activity arising from HH. Distinct baseline period (1–5 sec), impaired awareness period (11–17 sec), and automatism period (30–36 sec) are shaded in semi‐transparent light grey, purple, and pink, respectively. (G) Magnified representative LFPs inside HH (L‐pHH 1–2, grey star) and left hippocampus (L‐HIP 1–2, magenta star) during the three periods that are demonstrated in (F). (H) Frequency‐tracking cross‐coherence networks of the three periods. It shows the increased connectivity of various anatomical areas during different seizure semiology in the processing of ictal discharges. HH, hypothalamic hamartoma.

Differences have been observed regarding the involvement of extra‐HH structures in distinct types of clinical manifestations. One of the representative GS followed by a tonic seizure (patient 7) is shown in Figure 5A. Low‐amplitude fast activity occurred in the HH. Approximately a few seconds later, synchronized ictal discharges were observed between the HH and left orbitofrontal cortex ipsilateral to the predominant side of the hamartoma during the gelastic period. Nevertheless, quite different distributed areas were predominantly involved in the automatisms with FIAS. Figure 5B shows representative automatisms with FIAS in patient 5 with electrodes exploring the HH and cortical areas. Ictal discharges from the HH mainly propagated into mesial temporal structures including the amygdala and hippocampus as well as the temporal cortex during the impaired awareness period in the beta frequency band.

**Figure 6:**
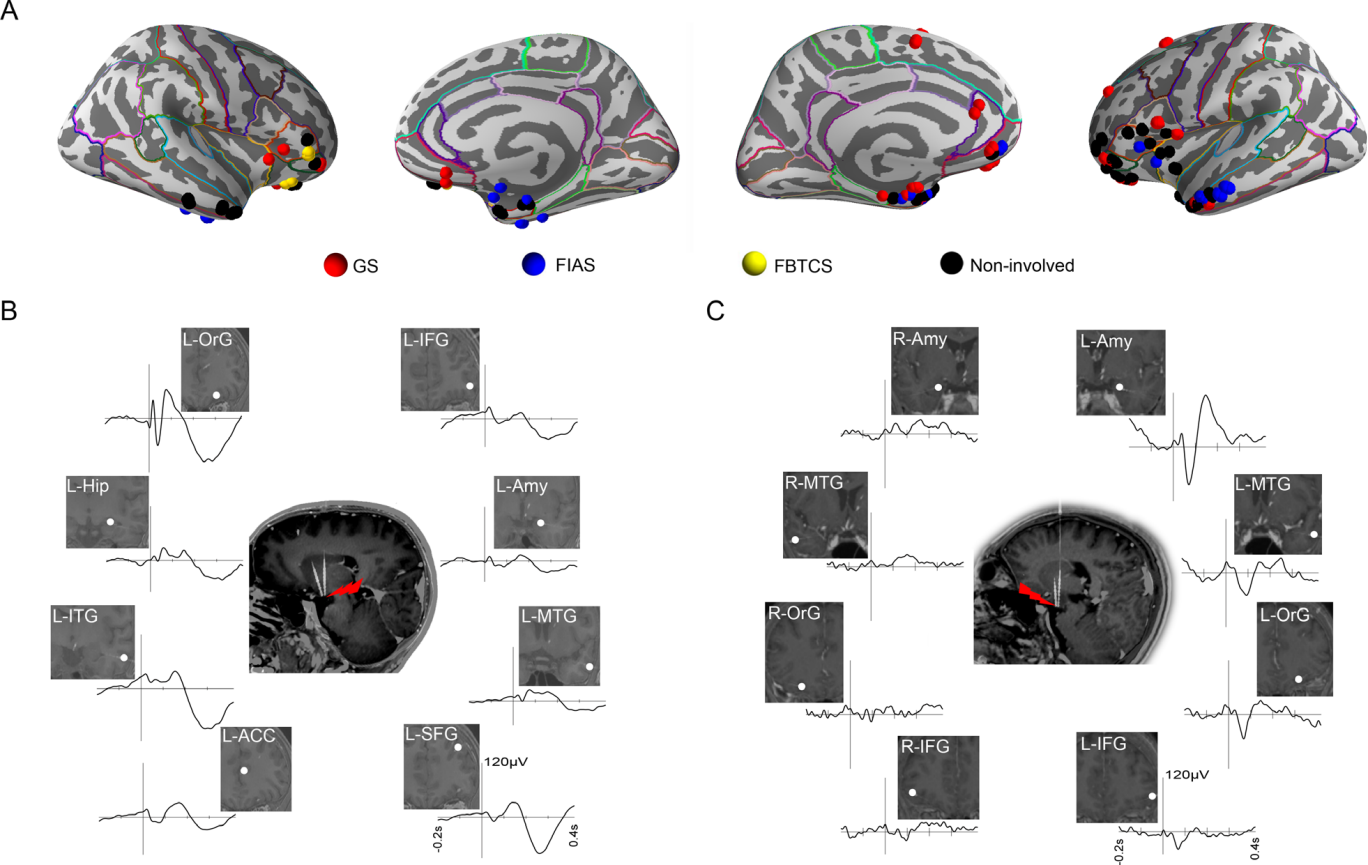
Overall cortical sites involving distinct seizures semiology (9 patients) and HH‐cortical evoked potentials (2 patients). (A) Each node corresponds to an electrode contact in the specified brain region with cortical parcellation using the Deskian/Killiany atlas. Each node corresponds to an electrode contact in the specified brain region. (B) HH‐cortical evoked potentials in patient 10 with prominent GS. (C) HH‐cortical evoked potentials in patient 9 with FIAS and GS. HH, hypothalamic hamartoma; GS, gelastic seizures; FIAS, focal impaired awareness seizure.

At the group level, the topological map showing the epileptogenic network of distinct seizure semiology of all 21 focal seizures from 9 patients with electrodes exploring cortical areas is indicated in Figure 6A. Clearly, GS propagated mainly through orbitofrontal areas, the cingulate gyrus and/or the neighboring limbic system, while automatisms with FIAS appeared with propagation mainly across the mesial temporal lobe.

The connectivity between the HH and cortex was evaluated using HH‐cortical evoked potentials in patients 9 and 10. With prominent GS, N1 component of averaged evoked potentials to HH stimulation was more remarkable in the orbitofrontal cortex than in the inferior frontal gyrus, amygdala and other distributed structures (Fig. 6B). In patient 9 with FIAS and GS, N1 component of averaged evoked potentials to HH stimulation was mainly detected in the amygdala ipsilateral to the attachment of the HH (Fig. 6C). These results were consistent with participation observed in distinct seizure networks.

### Epileptogenicity outside the limits of the HH and postoperative outcomes

**Figure 7:**
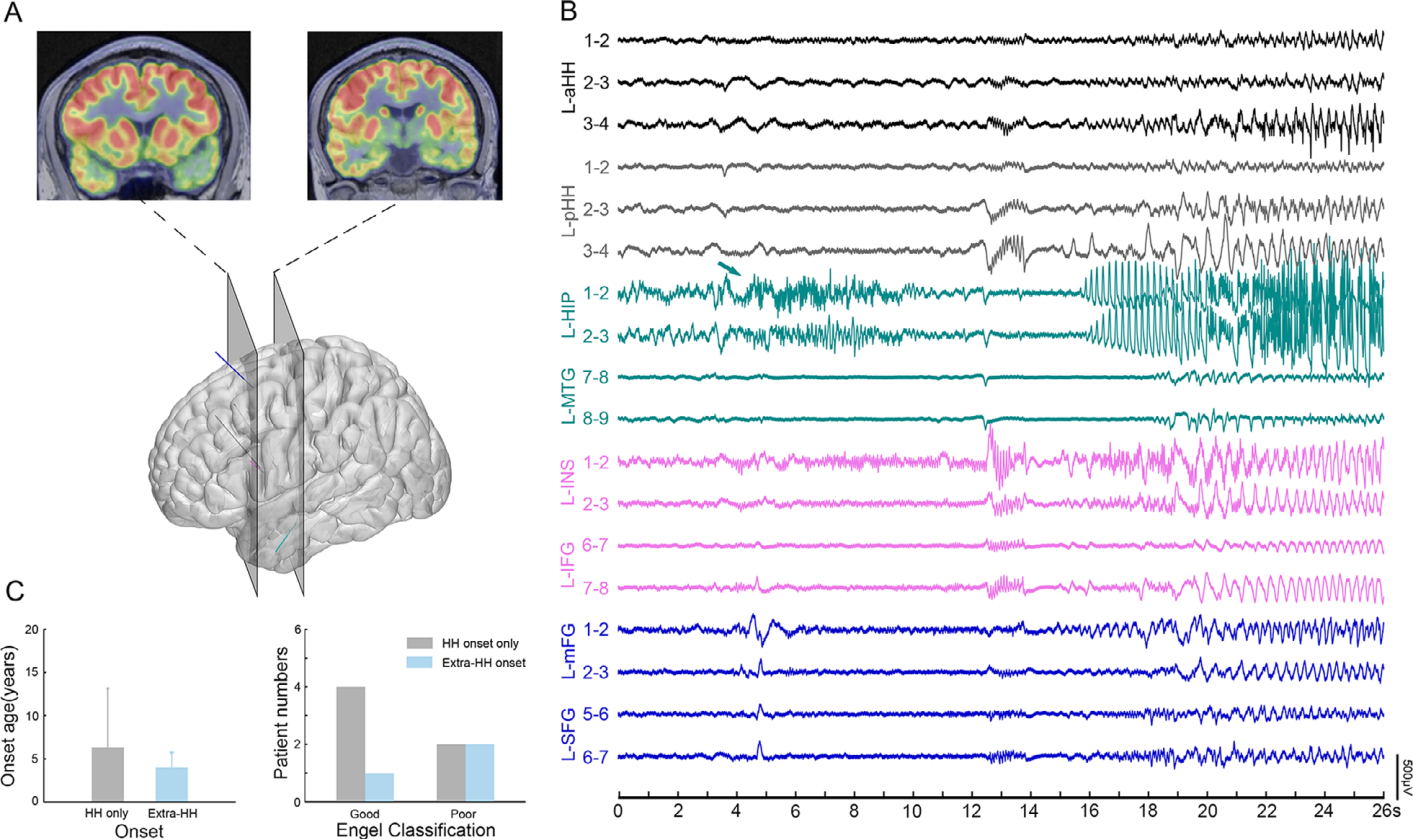
Independent extra‐HH seizure onset. (A) FDG‐PET show focal hypometabolism in left medial temporal lobe, insula, superior frontal lobe (Patient 11). (B) The green arrow indicates low amplitude fast activity arising from left hippocampus (Patient 11). (C) The comparison between seizure onset location (only HH onset and extra‐HH onset) and epilepsy onset age (left), and surgical outcome (right). Notably, good outcome refers to Engel class I and II while poor outcome refers to Engel class III and I. HH, hypothalamic hamartoma.

In this cohort, independent ictal discharges arising from the mesial temporal lobe were detected in three out of nine patients. Figure 7 shows an example with one seizure originating from the hippocampus but not the HH. Finally, only the HH was the target of the SEEG‐guided RF‐TC. Additionally, among these patients, it seemed that seizure onset originating from the HH only tended to be a potential predictor of good surgery outcomes compared with seizure onset originating from extra‐HH regions (mean follow‐up of 15.6 ± 11 months in these 9 patients; follow‐up time range of 15–36 months, as shown in Table S1).

## Discussion

We comprehensively study the intrinsic electrophysiological properties and seizure network of HH in a relatively large cohort of patients. First, we confirmed that different interictal biomarkers, including HFOs, can be detected within the HH. Subsequently, our study provides data relevant to the onset pattern of preictal discharge, ictal fast activity and simultaneous direct shift more specific to epileptogenic tissue itself. Furthermore, we have clearly shown the anatomo‐electroclinical correlations of various semiology in patients with HH. Finally, we revealed the extra‐HH‐independent ictal activity, which provided evidence of an extrahypothalamic secondary epileptogenic zone and might be a potential predictor of surgical outcomes.

In essence, epileptic tissue is characterized by paroxysmal electrical discharges. At the cellular level, Wu et al showed that small GABAergic inhibitory neurons predominantly in HH exhibit intrinsic “pacemaker‐like” firing behavior in a study of surgically resected tissue.^37^ At the local field potential level, the identification of the diverse patterns of interictal discharges, including HFOs that we first reported in vivo within the HH in the present study reflect an interplay of multiple distinct neuronal types within complex neuronal networks. In line with the findings from cortical lesions, our data favor the concept that distinct epileptogenic lesions share common interictal patterns across different pathologies. Interestingly, we have shown substantial synchronized and independent interictal epileptiform events between the HH and cortex, which are speculated to contribute to epileptic encephalopathy manifesting as disturbances of cerebral function in patients with HH.

As the ultimate expression of hypersynchronous neuronal activity arising from unbalanced runaway excitation, ictal discharge patterns have inherent implications for clinical significance.^38^ However, the question of the ictal pattern in the seizure‐onset zone in nature is still elusive. Traditionally, low‐amplitude beta‐gamma activity and its variations have been recognized as the main feature of the epileptogenic zone, but such ictal patterns may also emerge in propagated areas with some temporal delays.^39^ Otherwise, the ictal recordings from intra‐HH at broadband frequency that was revealed by our data were consistent with the uniform ictal onset pattern of the seizure‐onset zone, which was defined by three components, including quasiperiodic high amplitude preictal discharges, fast activity, and suppression of lower frequencies, suggesting an intrinsic feature of epileptic tissue.^40^

The epileptic seizure semiology associated with HH varies despite the unique seizure onset pattern.^41^ Seizure semiology is speculated to reflect the coordination among distributed brain areas that are functionally connected due to synchronized ictal discharges. By analyzing anatomo‐electroclinical correlations, our topographic map at the group level showed a possible epileptogenic network of distinct seizure semiology. The cingulate gyrus, neighboring limbic system and orbitofrontal cortex were preferentially affected in GS. Meanwhile, seizures with FIAS mainly result from the involvement of mesial temporal lobe structures. These preferred patterns of propagation have been further supported by direct evidence from the measurement of HH‐cortical evoked potentials.

In addition, there is an ongoing debate regarding the concept of kindling‐like secondary epileptogenesis responsible for the progressive evolution in patients with HH.^10^, ^42^ In this situation of possible secondary epileptogenesis, seizure outcomes were improved in two patients by additional temporal lobectomy as previously reported.^42^ This kind of independent temporal lobe origin has been subsequently described in one patient undergoing SEEG (patient 2 from Marseille).^10^ This patient was modeled using the Virtual Brain technology^43^ and complex seizure‐onset patterns were disclosed, where the HH was in the path of propagation. Other origins have also been reported, particularly those involving the frontal regions in tonic seizures (patient 1 from Marseille, patient 1–3 from Grenoble).^7^, ^8^, ^9^, ^10^ In the patient from London, tonic seizures were shown to originate from the HH instead of the frontal cortices.^4^ Consistent with previous reports,^10^ we also observed that other seizure types often appeared with a clear delay following the start of GS during the clinical course. Moreover, our data showed that independent epileptic ictal discharges arising from the mesial temporal lobe in three patients with HHs were thus largely suggestive of extended epileptogenicity outside the limits of the HH. Repetitive intrahamartoma interictal or ictal discharges are assumed to contribute to the development of the dependent or independent epileptogenesis on extra‐HH structures.

In summary, our data have comprehensively confirmed the interictal and ictal intrinsic electrophysiological properties of HHs in vivo. Our data also have revealed the preferential electroclinical network of seizure types. Taken together, the recognition of epileptogenic properties and the seizure networks associated with HHs might help to define and understand the abnormalities in epileptic tissue and epileptogenic networks.

## Author Contributions

L.K.R., Y.P.W., and G.G.Z. contributed to the conception and design of the study. G.G.Z., Y.Z.S., H.Q.Z., X.T.F., C.L., and Y.H.W. operated patients. D.W., Y.Z.S., Y.A., M.Y.L., and Y.F.Y. acquired the data. All authors contributed to the analysis of data. D.W., L.K.R., H.Q.Z., G.G.Z., J.L.D., S.Q.O., and P.H.W. contributed to preparing the figures, D.W., Y.Z.S., P.K., F.B., and Y.P.W. contributed to drafting the text.

## Conflict of Interest

We confirm that we have read the Journal’s position on issues involved in ethical publication and affirm that this report is consistent with those guidelines. None of the authors has any conflict of interest to disclose.

## Supporting information


**Table S1.** Clinical profiles of patients.
