# Supplementary material for: Electrophysiological properties and seizure networks in hypothalamic hamartoma
Source: Ann Clin Transl Neurol. 2020 Apr 16;7(5):653–66. doi: 10.1002/acn3.51033 (PMC7261749; doi:10.1002/acn3.51033)
Supplement: Supplementary file 1 — Table S1. Clinical profiles of patients. [file ACN3-7-653-s001.docx]

**Supplemental Table 1. Clinical profiles of patients**

| No. | Sex/  Age  (years) | Age at seizure onset (years) | | | Seizure frequency | Previous treatment | Trajectories of depth electrodes | Electrodes/  Contacts within HH | Electrodes/  Total contacts | Spontaneous seizure origination | | | Duration of follow-up (months) | Seizure outcome (Engel class) |
| --- | --- | --- | --- | --- | --- | --- | --- | --- | --- | --- | --- | --- | --- | --- |
|  |  | GS | FIAS | TCS |  |  |  |  |  | GS | FIAS | FBTCS |  |  |
| 1 | F/8 | 7 | - | - | 4-5/day | - | R-IFG → HH | 1/5 | 1/5 | HH | - | - | 50 | III |
| 2 | F/15 | 2^*^ | 13 | - | 2-6/day | - | R-MFG → aHH; R-MFG → pHH | 2/10 | 2/10 | HH | - | - | 49 | I |
| 3 | M/10 | 1 | - | 10 | 3-4/day | GKS & surgery | L-MFG → aHH; L-MFG → pHH | 2/10 | 2/10 | HH | HH | - | 45 | III |
| 4 | M/17 | 1 | - | 15 | 1/2 months | - | R-MFG → aHH; L-MFG → HH; R-MFG → pHH | 3/21 | 3/21 | - | - | HH | 45 | I |
| 5 | F/15 | 8 | 9 | - | 3-5/day | - | L-MFG → aHH; L-MFG → pHH; L-MTG → Amy;  L-MTG → HIP; L-IFG → CG; L-IFG → INS; L-SFG → INS | 2/24 | 7/78 | - | HH | - | 36 | I |
| 6 | M/33 | - | 19 | - | 2-3/week | - | L-MFG → HH; R-MFG → aHH; R-MFG → pHH; L-MTG → Amy; L-MTG → HIP; L-IFG → OrG; L-SFG → INS; R-MTG → Amy; R-MTG → HIP | 3/36 | 9/104 | - | HH | - |  |  |
|  | M/34 | - | 19 | - | 1-2/month | RF-TC | L-MFG → HH; R-MFG → HH | 2/16 | 2/16 | - | HH | - | 21 | III |
| 7 | F/8 | 6 | - | - | 2-5/day | - | R-MFG → aHH; R-MFG → pHH; L-IFG → OrG; R-IFG → OrG; R-IFG → INS; | 2/10 | 5/42 | HH | - | - | 27 | I |
| 8 | M/22 | 6 | 7 | 7 | 1-5/day | GKS | L-MFG → aHH; L-MFG → mHH; L-MFG → pHH; L-IFG → OrG; L-MTG → HIP; R-IFG → OrG; R-MTG → HIP | 3/15 | 7/63 | - | - | - | 26 | I |
| 9 | F/28 | 5 | 21 | - | 3-4/day | - | L-MFG → aHH; L-MFG → pHH; L-MTG → Amy; L-IFG → OrG; R-MTG → Amy; R-IFG → OrG | 2/16 | 6/60 | HH or L-Amy | - | - | 20 | III |
| 10 | M/19 | 3 | - | 13(always followed GS) | 1-2/day | GKS | L-MFG → aHH; L-MFG → pHH; L-MTG → Amy; L-MTG → HIP; L-MFG → OrG; L-SFG → CG | 2/16 | 6/62 | HH | - | - | 18 | II |
| 11 | M/22 | 2 | - | - | 2-3/day | - | L-MFG → aHH; L-MFG → pHH; L-MTG → HIP; L-IFG → INS; L-SFG → mFG | 2/16 | 5/42 | L-HIP | - | - | 17 | I |
| 12 | M/6 | 5 | - | 6 | 2-3/day | - | L-MFG → aHH; L-MFG → pHH; L-MTG → HIP; L-SFG → mFG; R-MTG → HIP | 2/16 | 5/48 | HH or L-HIP | - | - | 17 | III |
| 13 | F/9 | 1 | - | 2 | 1-2/day | surgery | L-MFG → HH; R-MFG → aHH; R-MFG → pHH; R-IFG → OrG | 3/24 | 4/36 | - | - | HH | 16 | III |
| 14 | M/19 | - | 9 | - | 1/2 months | - | L-MFG → aHH; L-MFG → pHH; R-MFG → aHH; R-MFG → pHH; R-MTG → HIP; R-IFG → OrG | 4/32 | 6/56 | - | - | - | 16 | I |
| 15 | M/37 | 1 | 14 | - | 1/2 weeks | - | L-MFG → aHH; L-MFG → pHH; R-MFG → aHH; R-MFG → pHH; L-MTG → HIP; L-IFG → OrG; R-MTG → HIP | 4/32 | 7/66 | HH | - | - | 15 | II |

^*^ The patient also had dacrystic seizure at the year before GS onset age which disappeared two years later.

F = female; M = male; GS = gelastic seizure; FIAS = focal impaired awareness seizure; FBTCS = focal to bilateral tonic–clonic seizure; GKS = Gamma Knife surgery; L = left; R = right; IFG = inferior frontal gyrus; MFG = medial frontal gyrus; SFG = superior frontal gyrus; ITG = inferior temporal gyrus; MTG = medial temporal gyrus; INS = insula; OrG = orbitofrontal gyrus; CG = cingulate gyrus; HH = hypothalamic hamartoma; aHH = anterior region of hypothalamic hamartoma; mHH = medial region of hypothalamic hamartoma; pHH = posterior region of hypothalamic hamartoma; Amy = amygdala; HIP = hippocampus.
